# Feasibility of Distributed Fiber Optic Sensor for Corrosion Monitoring of Steel Bars in Reinforced Concrete

**DOI:** 10.3390/s18113722

**Published:** 2018-11-01

**Authors:** Liang Fan, Yi Bao, Genda Chen

**Affiliations:** 1 Department of Civil, Architectural and Environmental Engineering, Missouri University of Science and Technology, Rolla, MO 65401, USA; lf7h2@mst.edu (L.F.); gchen@mst.edu (G.C.); 2 Department of Civil, Environmental and Ocean Engineering, Stevens Institute of Technology, Hoboken, NJ 07030, USA

**Keywords:** corrosion monitoring, distributed fiber optic sensor, electrochemical test, expansion strain, installation, pull-out test, steel mass loss

## Abstract

This study investigates the feasibility of distributed fiber optic sensor for corrosion monitoring of steel bars embedded in concrete. Two sensor installation methods are compared: (1) attaching the sensor along the bar and (2) winding the sensor on the bar. For the second method, optical fibers were winded spirally on steel bars with different spacings: 0 mm, 2 mm, 5 mm, and 10 mm. Steel bar pull-out testing was conducted to evaluate the effect of presence of distributed sensor on the bond strength of steel–concrete interface. Electrochemical testing was carried out to assess the influence of the installation methods on the corrosion resistance of the reinforced concrete. Winding the optical fiber on steel bars with a 10-mm spacing does not affect the bond strength and corrosion resistance and allows real-time corrosion monitoring. The distributed sensor data can be used to estimate the corrosion induced steel loss and predict concrete cracking.

## 1. Introduction

Corrosion is one of the major durability issues in reinforced concrete structures [1,2]. The high pH of concrete pore solution promotes the formation of a passive film on the surface of steel reinforcing bars for corrosion protection [3]. However, carbonation of concrete and ingress of chloride ions from ocean water or roadway deicing salt destroy the passive film, facilitating corrosion of steel bars [4,5]. In general, the corrosion products are expressed as *m*·Fe(OH)_2_ *n*·Fe(OH)_3_ *p*·H_2_O, where *m*, *n*, and *p* depend on the moisture and oxygen contents in the concrete [6]. The expansion of corrosion products exerts tensile stresses in the surrounding concrete matrix and causes concrete cracking. This in turn facilitates ingress of chloride ions, water and oxygen, accelerating the corrosion process. A theoretical, three-stage model was used to describe the corrosion-induced concrete cracking process [6]. First, in the free expansion stage, corrosion products diffuse to the capillary pores in the concrete matrix, without generating any stress in the matrix. Then, in the stress initiation stage, corrosion products fill up the pores at the steel–concrete interface and create increasing tensile stresses in the surrounding concrete matrix. Finally, in the cracking stage, the tensile stress exceeds the tensile strength of the matrix and causes concrete cracking. To date, there has been lack of in situ test data to verify the theoretical model. There is a need to develop effective monitoring tools.

Various corrosion sensors have been proposed for corrosion monitoring in reinforced concrete. The most widely used corrosion monitoring techniques are based on electrochemical methods, via measuring half-cell potential and linear polarization resistance (LPR) [7,8,9,10,11]. The measurement of half-cell potential is used to determine corrosion induced potential change, which can help detect occurrence of corrosion but cannot quantitatively assess corrosion condition [7,8]. The LPR method measures the polarization resistance, which is used to evaluate the corrosion rate via the Stern–Geary Equation [9]. However, the Stern–Geary Equation is only valid for uniform corrosion, and the measurement fails once separation of electrode from steel reinforcement occurs [10,11].

Fiber optic sensors are emerging tools for corrosion monitoring in recent years. Compared with the traditional electric corrosion sensors, fiber optic sensors carry numerous advantages such as high sensitivity, high precision, immunity to electromagnetic interference, etc. [12,13,14,15,16,17,18,19,20,21]. Fiber optic sensors can be categorized into point sensors and distributed sensors, in terms of their sensing length. Representative point corrosion sensors for reinforced concrete include fiber Bragg grating (FBG) and long-period fiber grating (LPFG) sensors. An FBG was attached on a polished steel bar to measure strain changes caused by corrosion [12]. An FBG temperature sensor composed of an FBG and carbon fiber strands was mounted to the surface of reinforcing bars before concrete casting [13]. The corrosion severity was tracked through the temperature change, because corrosion products have lower thermal conductivity compared with intact steel. However, the water content in the concrete and the bond condition of the sensor–steel interface can influence the sensitivity and precision of test data. A fiber optic sensor made of one FBG and twin steel bars was designed [14]. Each of the twin bars was split into two halves. The FBG sensor was placed in the middle of the two halves before packaged with concrete. The sensor was placed near the steel bar and the wavelength shift of the FBG sensor due to corrosion of the twin bars was used to detect corrosion of the steel bar. Another paradigm was to coat responsive materials on the cladding of the LPFG [15,16,17]. Corrosion of the coating material caused shift of the resonant wavelength of the LPFG; the wavelength shift was used to evaluate corrosion-induced mass loss of steel bar [15].

The above research showed great promise of the point corrosion sensors. However, since the corrosion condition varies in a structure, a large number of point sensors are needed to monitor a large-scale structure. In this sense, distributed fiber optic sensors are advantageous due to their detailed results and high spatial resolution [18,19,20,21]. The feasibility of distributed fiber optic sensor for corrosion monitoring was demonstrated [22,23]. Optical fibers were tightly winded on steel bars to measure the expansive strain caused by corrosion. Currently, there are at least two technical challenges that limit the application of the distributed corrosion sensor. First, the tightly winded optical fibers isolate the steel bars from the concrete, thus compromising the interfacial properties. It is likely that both the bond strength and corrosion resistance are compromised, because the isolation also hinders the formation of the passive film on the bar surface. Second, corrosion can hardly be located, given the spatial resolution down to meter order for the conventional distributed sensing technologies. There is a need to develop a corrosion monitoring technique with a high spatial resolution. Further study is needed to understand the effect of sensor installation on the bond strength and corrosion resistance.

This study aims to address the above two challenges through experimental investigations. A pulse pre-pump Brillouin optical time domain analysis (PPP-BOTDA) technology is used to measure corrosion induced strain changes with a spatial resolution of 2 cm. Two different sensor installation methods are experimentally investigated. The first method is to attach the fiber along the bar; the second method is to wind the fiber on the bar. For the second method, optical fibers were winded spirally on steel bars with different spacings: 0 mm, 2 mm, 5 mm, and 10 mm. Pull-out testing was conducted to evaluate the effect of the installation methods on the interfacial property of reinforced concrete. Electrochemical testing was carried out to assess the influence of the installation methods on the corrosion resistance of reinforced concrete. This study develops a real-time corrosion monitoring tool for reinforced concrete. The tool can be useful for condition monitoring of large-scale civil infrastructure.

## 2. Distributed Fiber Optic Sensor

### 2.1. Working Principle

When a light wave travels in an optical fiber, the variation of the electric field of the light generates acoustic waves through electrostriction, periodically altering the refractive index of the fiber. In a Brillouin optical time domain analysis (BOTDA), a pump pulse and a probe continuous wave are launched from the two ends of an optical fiber and counter propagate along the fiber. When the frequency difference of the two waves match the Brillouin frequency of the fiber, Brillouin gain occurs, thus increasing the signal-to-noise ratio. The Brillouin frequency is associated with the strain and temperature of the fiber, and thus can be used to measure strain and temperature [24,25]. Compared with the conventional BOTDA, PPP-BOTDA uses a long-duration pre-pump pulse to stimulate phonon prior to a short-duration pulse [18]. The duration of the pre-pump pulse must be longer than the life time of phonon (~10 ns) [26]. Since the spatial resolution is proportional to the pulse width, a narrow pulse (0.2 ns) is used to achieve a high spatial resolution (2 cm). At normal temperature, the Brillouin frequency shift (Δν*_B_*) is linked with the temperature and strain changes using a linear equation:

(1) $$\Delta v_{B}=C_{\varepsilon }\Delta \varepsilon +C_{T}\Delta T$$

where *C_ε_* and *C_T_* are the strain and temperature sensitivity coefficients, respectively; Δε and ΔT are the strain and temperature changes, respectively. In this study, experiments were conducted at constant room temperature (22 °C). Thus, Brillouin frequency shift was caused by strain change.

### 2.2. Optical Fiber

**Figure 1:**
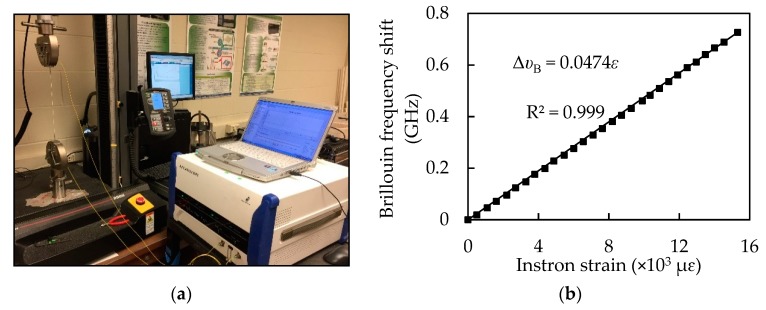
Uniaxial tensile testing for calibration of the fiber optic sensor: (**a**) laboratory test set-up; (**b**) sensor calibration curve.

Telecommunication-grade single-mode optical fibers were used as distributed sensors [24,25,26]. Each fiber consisted of a core (diameter: 8.2 µm), a cladding (diameter: 125 µm), an inner coating (diameter: 190 µm), and an outer coating (diameter: 242 µm). The fiber core and cladding were made of high-purity silica; the inner and outer coatings were made of acrylic for mechanical protection. The strain sensitivity coefficient of the sensor was calibrated using the method in [19]. Three optical fibers with a gauge length of 250 mm were tested in tension at a displacement rate of 0.16 mm/min using a low-capacity load frame (model: Instron 5965, load capacity: 100 N) and a Neubrescope at room temperature (22 °C), as depicted in Figure 1a. Each optical fiber was passed through two protective sleeves that had a steel rod measuring 1.5 mm in diameter for mechanical protection. The protective sleeves were then bonded to the ends of the optical fibers and gripped by the load frame fixture for testing. The applied load and the fiber elongation were recorded by Instron at a sampling frequency of 10 Hz. Given the initial length of optical fiber, the strain values can be calculated from the elongation. At each load, the strain in the optical fiber was simultaneously measured using the load frame and the Neubrescope. The Neubrescope was used to measure the Brillouin frequency shift that can be converted into the strain change from Equation (1) when tested in the ambient temperature. Figure 1b plots the Brillouin frequency shift against the tensile strain of the optical fiber. The coefficient of determination (R^2^) for the linear regression is 0.999, indicating a good correlation. The strain sensitivity coefficient is represented by the slope of the line with the value of 4.74 × 10^−5^ GHz/με.

## 3. Materials

### 3.1. Concrete

A Type I ordinary Portland cement was used. The chemical composition was determined using the X-ray flourescense (XRF) and quantitative X-ray diffraction (QXRD) techniques, as listed in Table 1 and Table 2, respectively.

Table 3 lists the mix proportion of the concrete. The water-to-cement ratio was 0.5. After concrete casting, the specimen was covered with wet burlap and plastic sheet to prevent surface cracking due to drying shrinkage. The specimen was demolded after 24 h, and then cured in an environmental chamber for 28 days. The temperature and relatively humidity in the environmental chamber were 22 ± 2 °C and 90 ± 5%, respectively. The compressive strength and tensile strength of each type of concrete were evaluated in accordance with ASTM C39 [27] and ASTM C496 [28]. Five cylinders were prepared for each test. The average compressive and tensile strengths at 28 days were 37 ± 1.1 MPa (mean value ± standard deviation) and 2.5 ± 0.1 MPa, respectively.

### 3.2. Steel Bars

Two types of deformed steel bars were used: the first type with a diameter of 19.1 mm for the corrosion tests, and the other type with a diameter of 12.7 mm for the pull-out test. The chemical composition is listed in Table 4. The minimum requirements of the yield strength, ultimate strength, and elongation are 420 MPa, 620 MPa, and 9%, respectively, as specified by ASTM A615 [29]. The manufacturer specified Young’s modulus is 205 GPa.

## 4. Experimental Study

### 4.1. Pull-Out Test

**Figure 2:**
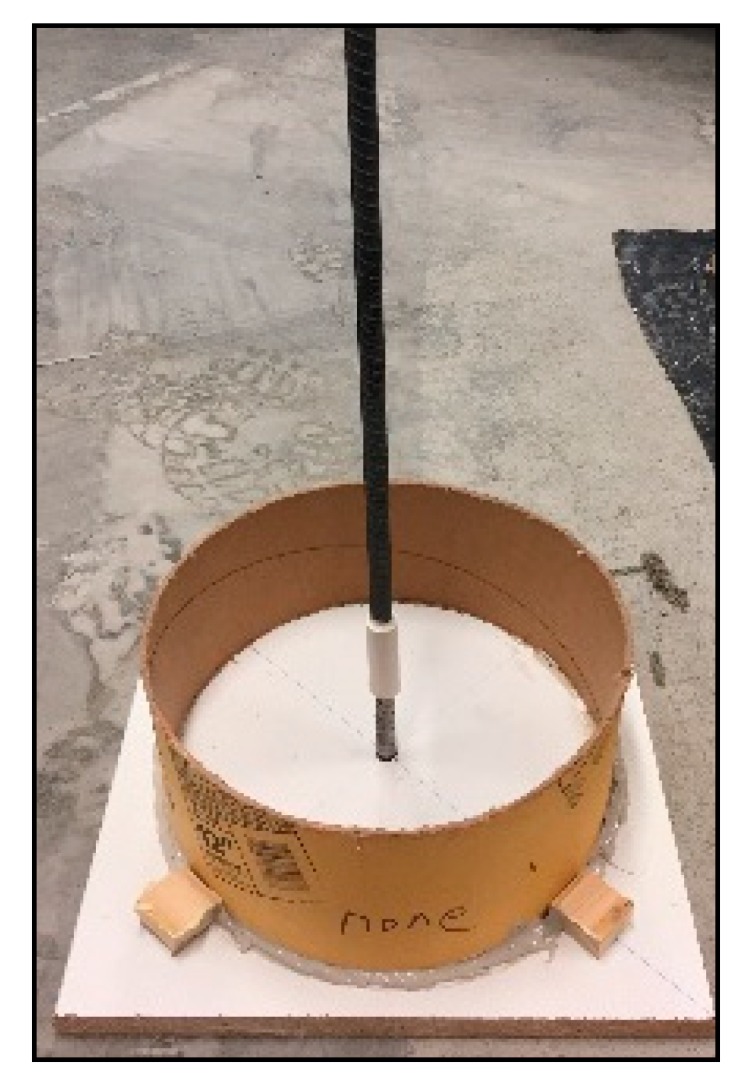
Pull-out test specimen.

The pull-out specimens were designed in compliance with RILEM-II-128 [30] and modified to ensure the specimens failed in the pull-out pattern. Each cylinder measured 305 mm in diameter and 128 mm in length. In each cylinder, a half of the embedded length of the steel bar was in direct contact with the concrete, while the other half was isolated using a polyvinyl chloride (PVC) tube [31], as shown in Figure 2.

**Figure 3:**
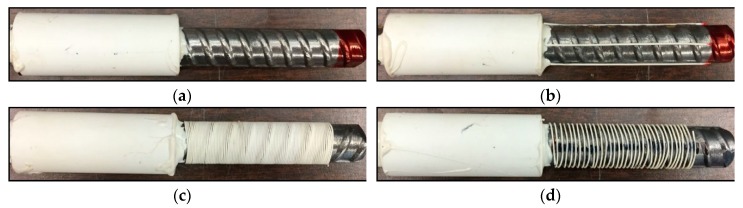

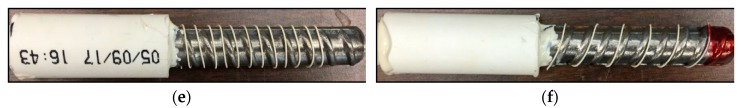
Photograph of steel bars in concrete cylinders for pull-out test: (**a**) Ref; (**b**) L4; (**c**) S0; (**d**) S2; (**e**) S5; (**f**) S10.

Figure 3 shows the steel bars with optical fibers. The specimen without any fiber on the bar is taken as the control specimen, designated as Ref (Figure 3a); the specimen with four fibers equally spaced along the bar is designated as L4 (Figure 3b); the specimens with spirally winded fibers are designated as S0, S2, S5, and S10, corresponding to the spacing of 0 mm, 2 mm, 5 mm, and 10 mm, respectively, as shown in Figure 3c,f. The PVC tube that covers a half of the embedded bar length is the bond breaker. Styrofoam strips were installed on the bars to ensure the bar was centered in the PVC tube. The bond breaker was fixed on the bar using a two-part epoxy at the ends of the concrete cylinder.

**Figure 4:**
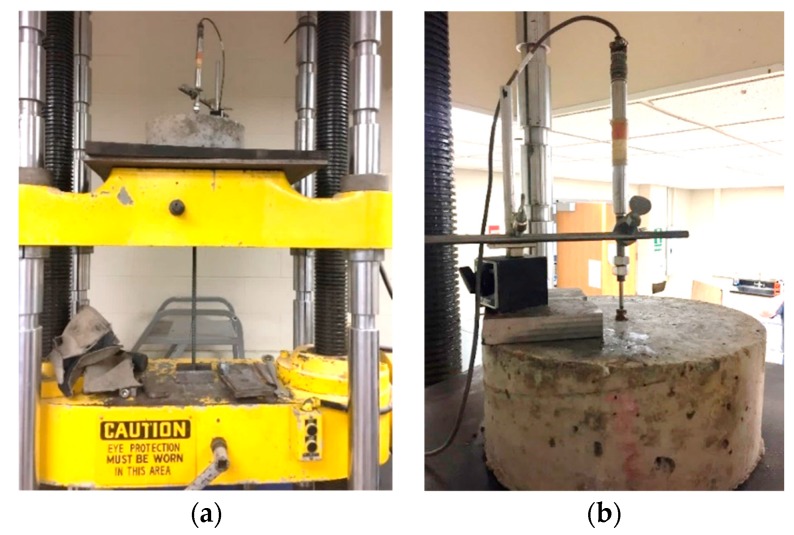
Pull-out test: (**a**) loading set-up; and (**b**) instrumentation.

The pull-out specimens were tested using a load frame (capacity: 890 kN) at a constant loading rate of 2.5 mm/min, as shown in Figure 4. The bar was passed through a neoprene pad (20 mm thick), a steel plate (10 mm thick) and the load frame, and gripped by the fixture of the load frame (Figure 4a). The steel plate was used to provide support to the concrete base; the neoprene pad was used to alleviate stress concentration. A linear variable differential transformer (LVDT) was used to measure the relative slip between the bar and concrete, as shown in Figure 4b.

### 4.2. Corrosion Test

**Figure 5:**
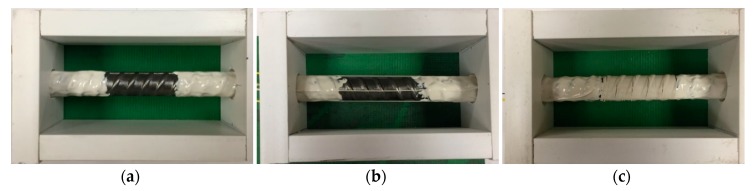

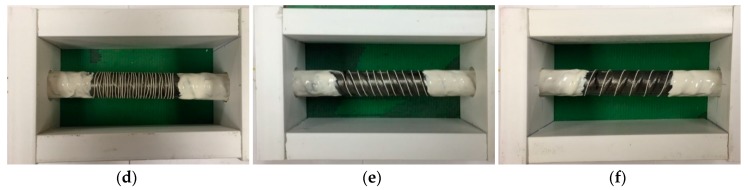
Steel bars in the beams for corrosion testing: (**a**) Ref; (**b**) L4; (**c**) S0; (**d**) S2; (**e**) S5; (**f**) S10.

Reinforced concrete beams measuring 140 mm in length with a 70 mm by 70 mm square cross section were prepared for corrosion test, as depicted in Figure 5. The steel bar was 180 mm long, and had a nominal diameter of 19.1 mm. The surfaces of steel bars were sand blasted to remove the rust and then cleaned using alcohol. A copper wire was soldered at each end of the steel bar for accelerated corrosion testing and electrochemical test measurement. The installation of optical fibers on the bars was consistent with that for the pull-out test, as shown in Figure 5b–f. A two-part epoxy was used to cover a 60-mm length of the bar at each end to enforce electrochemical reactions within the middle length of the bar. Different specimens had different fiber lengths, due to the different fiber installation methods and different spacing. In this research, the length of fiber installed on the steel bars varied from 0.2 m to 2.9 m; the total fiber length was in the range of 0.7 m to 3.4 m.

Electrochemical tests and accelerated corrosion test were conducted using the reinforced concrete beam specimens at 28 days. Before the corrosion test, a low-viscous, two-part epoxy was used to seal the other surfaces of the beam to minimize water transport, except for the top surface. The open circuit potential (OCP) was measured using the beam specimens in 3.5 wt.% sodium chloride solution until the OCP became stabilized. After that, a constant current of 500 µA/cm^2^ was impressed through all the specimens to accelerate corrosion. The accelerated corrosion was paused every 2 h to evaluate the OCP and LPR, and measure the strain distributions using the distributed sensors. The specimens were tested at room temperature with a standard three-electrode setup, including a graphite rode measuring 6 mm in diameter as the counter electrode, a saturated calomel electrode as the reference electrode, and the steel bar as the working electrode. All three electrodes were connected to a potentiostat (model: Gamry Interface 1000E) for data acquisition. After each stable OCP was recorded, the LPR was evaluated within 15 mV around the OCP at a rate of 0.167 mV/s. The polarization resistance (*R_p_*) is the slope of the linear region of a polarization curve around zero current [32]:

(2) $$R_{p}=\Delta E/\Delta I$$

where ∆*E* and ∆*I* represent the voltage and current increments at *I* = 0, respectively.

### 4.3. Corrosion Test

In the accelerated corrosion test, the steel bar and a graphite rod were respectively connected to the positive and negative electrodes of the power supply. A circuit between the steel bar and graphite rod was conducted by transport of ions when the constant current was applied [33]. In this study, the moment when the mass loss of the steel bar reaches 2% is defined as completion of the corrosion test.

## 5. Results and Discussion

### 5.1. Pull-Out Test

**Figure 6:**
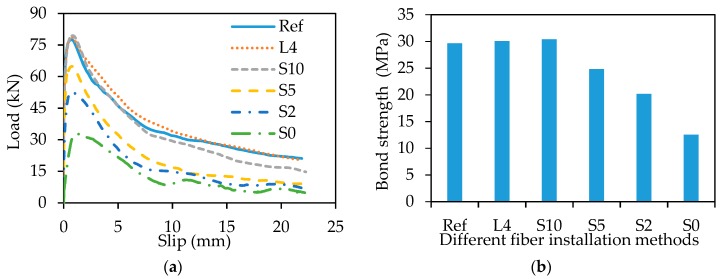
Pull-out test result: (**a**) load-slip curves; (**b**) bond strength.

Figure 6a shows the load-slip curves. Each curve is composed of three parts. First, the applied load approximately linearly increases with the slip. Then, the applied load increases with the slip at a reduced increasing rate until reaching the peak. Finally, the load decreases with the slip until the bar is pulled out. The bond strength is calculated using [31]:

(3) $$\tau =\frac{P}{\pi d_{b}l_{d}}$$

where *τ* is the bond strength; *P* is the peak load; *l_d_* is the embedded length; *d_b_* is the bar diameter.

No significant difference was observed from the load-slip curves and the bond strengths of the specimens Ref, L4, and S10, indicating that the fiber installation of L4 and S10 do not significantly affect the bond strength. However, as the spacing is reduced from 10 mm to 5 mm, the bond strength is reduced by 18% (Figure 6b).

### 5.2. Electrochemical Test

**Figure 7:**
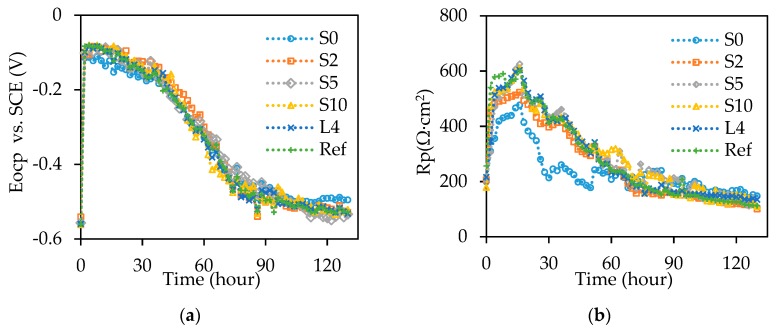
Electrochemical test results of: (**a**) OCP, and (**b**) *R_P_*. SCE stands for saturated calomel electrode.

Figure 7a,b respectively show that the OCP and *R_P_* results of all the reinforced concrete beams follow the similar trends over the time up to 130 h. Before the accelerated corrosion test, the OCP is around −0.55 V. After the accelerated corrosion test for 2 h, the OCP increases to around −0.10 V, followed by a slow decrease to −0.15 V at 36 h. Then, the OCP approximately linearly decreases to −0.45 V at 72 h. Finally, the OCP decreases at a smaller rate until the end of the test. Except for the specimen S0 of which OCP stabilizes at −0.50 V, the OCPs of the other specimens stabilized around −0.53 V.

Before the accelerated corrosion test, the *R_P_* values of all the specimens are lower than 250 Ω·cm^2^. After the accelerated corrosion test for 2 h, *R_P_* of the specimens S0, S2, S5, S10, L4, and Ref rapidly increases to 309 Ω·cm^2^, 450 Ω·cm^2^, 305 Ω·cm^2^, 475 Ω·cm^2^, 344 Ω·cm^2^, and 540 Ω·cm^2^, respectively; at 16 h, *R_P_* of the specimens S0, S2, S5, S10, L4, and Ref gradually increases to 476 Ω·cm^2^, 522 Ω·cm^2^, 625 Ω·cm^2^, 611 Ω·cm^2^, 603 Ω·cm^2^, and 600 Ω·cm^2^, respectively. After that, the *R_P_* values decrease quickly to 210 Ω·cm^2^, 170 Ω·cm^2^, 263 Ω·cm^2^, 231 Ω·cm^2^, 184 Ω·cm^2^, and 190 Ω·cm^2^ for the specimens S0, S2, S5, S10, L4, and Ref, respectively, until 68 h, followed by a linear reduction until completion. Compared with the other specimens, S0 has relatively lower *R_P_* values from 0 to 60 h and higher values after 60 h. The different OCP and *R_P_* values indicate that the corrosion process is associated with the sensor installation method.

The increase of OCP and *R_P_* values at the beginning are attributed to the accumulation of corrosion products in concrete pores, which increase the concrete resistance [7,34]. After the corrosion products filled the concrete pores, the corrosion products accumulated at the interface between steel and concrete. As concrete confined the expansion of corrosion products, the corrosion products are compacted and densified on the steel surface, which inhibits the transport of oxygen and decreases the OCP and *R_P_* values [35].

### 5.3. Distributed Fiber Optic Sensor Measurement

**Figure 8:**
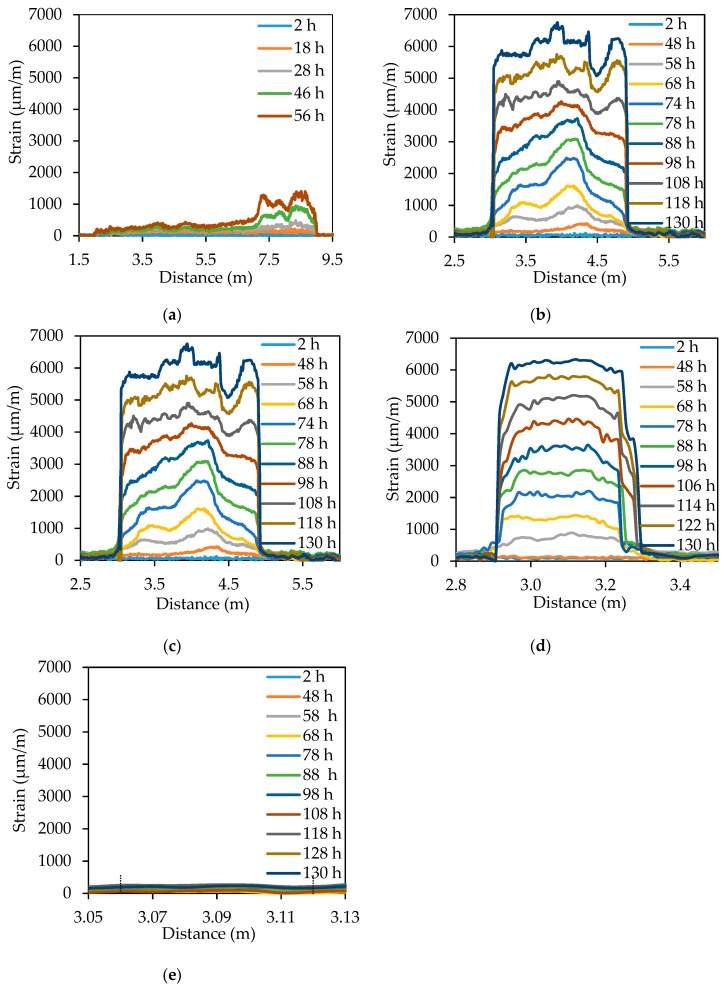
Detailed strain distributions along the distributed fiber optic sensor for different installation methods: (**a**) S0; (**b**) S2; (**c**) S5; (**d**) S10; and (**e**) L4.

Figure 8 shows the strain distributions measured from the distributed sensors installed in the reinforced concrete beams. The *X*-axis represents the distance along the fiber length; the zero distance is at the pump end of the data acquisition system. The *Y*-axis represents the strain measured from the distributed sensor; tensile strain is positive. Each figure displays the strain in the length of the fiber winded on the steel bar and a short length of the fiber at each of its two ends.

Figure 8a shows the strain results from S0. There is no obvious strain change within the first 18 h. Then, the strain increases with time until 56 h. The strain change within the length 7.2 m to 9.0 m is larger than the strain change in the other parts of the fiber, indicating that corrosion of the bar length that corresponds to the fiber length from 7.2 m to 9.0 m is more severe. After 56 h, the distributed sensor failed to measure, because of significant signal loss caused by the corrosion induced macrobend of the optical fiber. The macrobend loss is dependent on the bend radius and number of circles. Thus, S0 is more susceptible to macrobend loss compared with the other specimens. Figure 8b shows the strain distributions from S2. The fiber length from 3.0 m to 5.0 m is subjected to strain changes that increase over time until 118 h. The distributed sensor continued to be functional and provided reasonable measurement after 130 h. Once again, non-uniform strain distributions are observed at each time instant. Similarly, Figure 8c,d show the strain distributions from S5 and S10, respectively. The distributed sensors in S5 and S10 continued to be functional after 130 h. Figure 8e shows the strain results from L4. The fiber length from 3.06 m to 3.12 m was in contact with the steel bar. There was negligible strain change over 130 h, revealing that the distributed sensor in L4 failed to detect corrosion of the bar.

**Figure 9:**
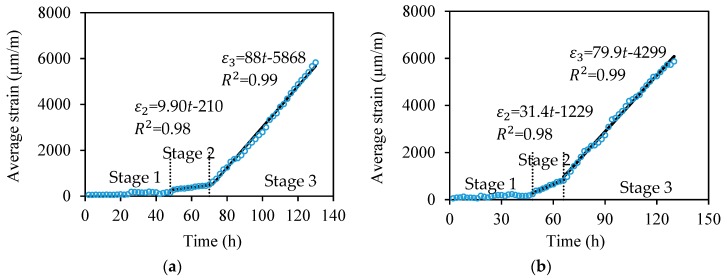

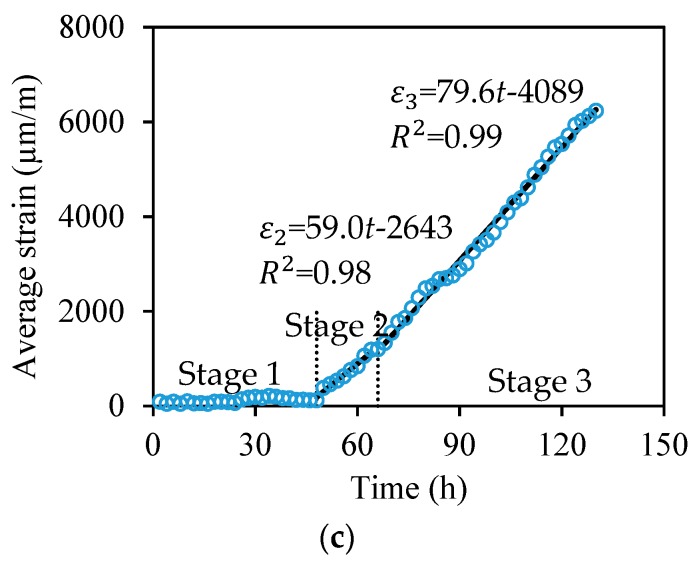
Relationships between the average strain (*ε*) and corrosion test time (*t*) of (**a**) S2, (**b**) S5, and (**c**) S10, show three stages (Stages 1, 2, and 3); in Stages 2 and 3, *ε* approximately linearly increases with *t*.

To investigate the strain increasing rate in different specimens, the expansion strain measured from the distributed sensor is averaged over the fiber length in contact with the steel bar at each time instant. Figure 9a–c plot the averaged strains of S2, S5, and S10, respectively. Each curve can be divided into three stages. In Stage 1, the corrosion products diffuse and gradually filled the surrounding concrete pores [7,34]. This is corroborated by the very small strain change (less than 150 με) measured from the distributed sensors. Stage 1 is terminated when the majority of concrete pores at the steel–concrete interface is filled up and the corrosion products starts to increase the tensile strains. In Stage 2, the corrosion products continue to expand and tend to increase the tensile stresses in the concrete matrix. Stage 2 ends when concrete cracks. In Stage 3, ingress of water and oxygen is accelerated due to the concrete cracking [6,20]. Thus, the average strain increases approximately linearly with time at an increasing rate larger than that of the Stage 2. A linear regression analysis is conducted for Stage 2 and Stage 3, respectively. For the specimens S2, S5, and S10, the strain increasing rate of Stage 2 is 9.9 με/h, 31.4 με/h, and 59.0 με/h, respectively; the strain increasing rate of Stage 3 is 88 με/h, 79.9 με/h, and 79.6 με/h, respectively. 

Typically, corrosion of a steel bar is uniform along the bar length when the bar is impressed with a constant current. In this test, the strain distributions measured from S0, S2, and S5 are non-uniform along the bar; the strain distributions measured from S10 are more uniform. This indicates that the fiber wrapping spacing influences the corrosion pattern. A small fiber spacing tends to cause non-uniform distribution of corrosion. 

In Stage 2, the strain increasing rate decreases with the spacing, because the tightly spaced fibers occupy more space outside of the steel bars, hindering ingress of oxygen and water to the bars, thus decelerating the corrosion rate. In Stage 3 when the concrete cracks, ingress of oxygen and water is largely promoted by the cracks, and thus the effect of fiber spacing becomes less predominant. Therefore, the strain increasing rates of S2, S5, and S10 are close in Stage 3.

In this study, no additional mechanical loading was applied to the tested specimens. In real-world civil engineering applications, reinforced concrete is subjected to mechanical loading, which also influences the strain in the distributed sensors [18]. It is envisioned that additional sensors are needed to compensate the effect of mechanical loading and temperature on the measurement results. Further research is needed to test the performance of the distributed corrosion sensors using reinforced concrete specimens under mechanical loading and understand the effect of mechanical loading on corrosion process. 

### 5.4. Quantification of Corrosion Induced Volume Expansion

The mass loss (Δ*m*) of the steel bar per unit length (1 cm in this case) is calculated [36]:

(4) $$\Delta m=\frac{WIt}{nF}$$

where *W* is the atomic weight of iron (56 g/mol); *I* is corrosion current in each bar (*I* = 2π*r*_0_·*i*·*l*); i is corrosion current density (500 µA/cm^2^); *r*_0_ is the radius of the bar before corrosion (9.55 mm); *l* equals to 10 mm in this case; *t* is the duration of the applied current; *n* is the ionic charge (*n* = 2 for iron); *F* is a constant (96,500 A·s/mol). 

The mass loss of the steel bar in each stage can be expressed as:

(5) $$\Delta m=\rho \cdot \pi r_{n-1}^{2}-\rho \cdot \pi r_{n}{}^{2}$$

Thus, the radius of the corroded steel bar excluding the rust layer is:

(6) $$r_{n}={\left(r_{n-1}^{2}-\frac{\Delta m}{\rho \cdot \pi }\right)}^{0.5}$$

where *ρ* is the density of steel (7.86 g/cm^3^); *n* = 1, 2, and 3 for Stage 1, Stage 2, and Stage 3, respectively. The radius of the steel bar with rust is:

(7) $$r_{c}=r_{0}(1+\varepsilon_{t})$$

where $\varepsilon_{t}$ is the expansion strain of the corroded steel bar measured from the distributed sensor. 

**Figure 10:**
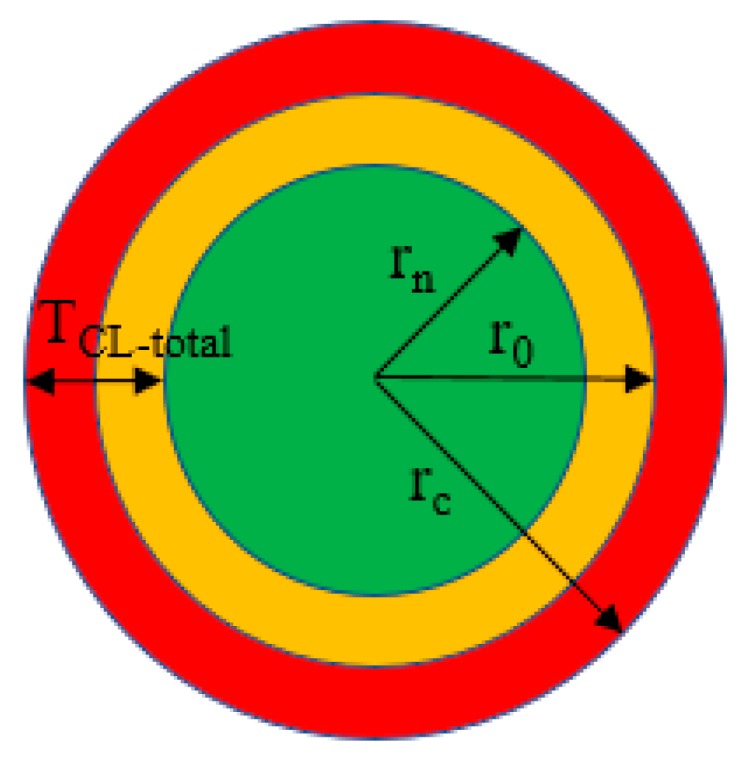
Schematic of the radius change of steel bar under corrosion.

The total thickness of the corrosion layer is denoted by *T_CL-total_* as shown in Figure 10 [23]:

(8) $$T_{CL-total}=r_{c}-r_{n}$$

The total volume of the corrosion layer (*V_CL-total_*) is:

(9) $$V_{CL-total}=\pi \cdot 1\cdot [r_{c}{}^{2}-r_{n}^{2}]$$

Based on Equations (4)–(9), the radius of the corroded steel bar, the corrosion product layer thickness and volume are determined, as listed in Table 5. *T_CL-total_* and *V_CL-total_* can be used to predict concrete cracking. The different sensor installation methods do not show signicant influence on *T_CL-total_* and *V_CL-total_*. The specimen S10 is selected for discussion. At the end of Stage 1, *T_CL-total_* is 0.15 mm, larger than the radius loss of the steel bar (0.032 mm). At the end of Stage 2, the steel mass loss reaches 0.92%; *T_CL-total_* is 1.131 mm. By the end of Stage 3, the steel mass loss reaches 2%; *T_CL-total_* is 5.909 mm, which is more than five times that of Stage 2.

## 6. Conclusions

Based on the above investigations, the following conclusions can be drawn:

- Spirally winding the optical fiber on steel bar with a spacing of 10 mm does not compromise the bond strength of steel–concrete interface and the corrosion resistance of reinforced concrete beams. This indicates that the installation method of the distributed fiber optic sensor is plausible for corrosion monitoring of reinforced concrete.
- According to the distributed sensor data, the corrosion process can be divided into three stages in terms of the strain increasing rate, thus experimentally verifying the three-stage theory. In Stage 1, the corrosion induced strain change is small (within 200 με). In Stage 2 and Stage 3, the strain approximately linearly increases with time. The strain increasing rate is higher in Stage 3 than that in Stage 2 due to the presence of concrete cracks.
- Using a winding spacing up to 5 mm reduces the bond strength of steel-concrete interface by 18%, and thus compromises the mechanical property of reinforced concrete. Distributed sensors along the steel bar is insensitive to the corrosion of the bar.
- The strain distributions measured from the distributed sensor can be used to quantify the corrosion condition. The corrosion layer thickness and volume can be estimated using the measured strain distributions.
- Further research is needed to test the performance of the proposed corrosion monitoring method using reinforced concrete under mechanical loading, and understand the effect of mechanical loading on the corrosion process.

## Figures and Tables

**Table 1:** Mass percentage of oxides determined by XRF for the cement.

SiO_2_	Al_2_O_3_	Fe_2_O_3_	CaO	MgO	SO_3_	Loss of Ignition
19.8	4.5	3.2	64.2	2.7	3.4	2.6

**Table 2:** Mass percentage of crystalline phases determined by QXRD for the cement.

Alite (C_3_S)	Belite (C_2_S)	Aluminate (C_3_A)	Ferrite (C_4_AF)	Gypsum (C$.2H)
3.2	64.2	2.7	3.4	2.6

**Table 3:** Concrete mixture proportion.

Water	0.5
Ordinary Portland cement	1.0
Missouri river sand	2.0
Coarse aggregate	2.5

**Table 4:** Chemical composition of the steel bars.

Element	C	Si	Mn	P	S	Cr	Mo	Ni	Co	Cu	V	Sn	Fe
Wt.%	0.38	0.18	1.00	0.12	0.06	0.10	0.07	0.20	0.01	0.37	0.02	0.03	97.40

**Table 5:** Corrosion induced volume expansion.

Designation	Stage	Duration (h)	Δ*m* (g)	*r_n_* (mm)	*r*_0_–*r_n_* (mm)	*T_CL-total_* (mm)	*V_CL-total_* (mm^3^)
S2	1	48	0.15	9.518	0.032	0.202	122
	2	18	0.05	9.506	0.044	0.818	510
	3	64	0.20	9.465	0.085	5.894	4594
S5	1	48	0.15	9.518	0.032	0.177	107
	2	18	0.05	9.506	0.044	0.845	527
	3	64	0.20	9.465	0.085	5.854	4556
S10	1	48	0.15	9.518	0.032	0.150	91
	2	18	0.05	9.506	0.044	1.131	716
	3	64	0.20	9.465	0.085	5.909	4608
